# Galectin-3 in septic acute kidney injury: a translational study

**DOI:** 10.1186/s13054-021-03538-0

**Published:** 2021-03-18

**Authors:** Haibing Sun, Huiping Jiang, Amity Eliaz, John A. Kellum, Zhiyong Peng, Isaac Eliaz

**Affiliations:** 1 Department of Critical Care Medicine, Zhongnan Hospital, Wuhan University, Wuhan, Hubei Province China; 2 School of Medicine, University of California, San Francisco, CA USA; 3 Center of Critical Care Nephrology, Department of Critical Care Medicine, University of Pittsburgh Medical Center, Pittsburgh, PA USA; 4 Amitabha Medical Center, Santa Rosa, CA USA

**Keywords:** Galectin-3, Sepsis, Acute kidney injury

## Abstract

**Background:**

Galectin-3 (Gal-3) is a pleiotropic glycan-binding protein shown to be involved in sepsis and acute kidney injury (AKI). However, its role has never been elucidated in sepsis-associated AKI (S-AKI). We aimed to explore Gal-3’s role and its potential utility as a therapeutic target in S-AKI.

**Methods:**

In 57 patients admitted to the intensive care unit (ICU) with sepsis, serum Gal-3 was examined as a predictor of ICU mortality and development of AKI. In a rat model of S-AKI induced by cecal ligation and puncture (CLP), 7-day mortality and serum Gal-3, Interleukin-6 (IL-6), and creatinine were examined at 2, 8, and 24 hours (h) post-CLP. Two experimental groups received the Gal-3 inhibitor modified citrus pectin (P-MCP) at 400 mg/kg/day and 1200 mg/kg/day, while the control group received water only (*n* = 18 in each group).

**Results:**

Among 57 patients, 27 developed AKI and 8 died in the ICU. Serum Gal-3 was an independent predictor of AKI (OR = 1.2 [95% CI 1.1–1.4], *p* = 0.01) and ICU mortality (OR = 1.4 [95% CI 1.1–2.2], *p* = 0.04) before and after controlling for age, AKI, and acute physiology and chronic health evaluation (APACHE II) score. In the CLP rat experiment, serum Gal-3 peaked earlier than IL-6. Serum Gal-3 was significantly lower in both P-MCP groups compared to control at 2 h post-CLP (400 mg: *p* = 0.003; 1200 mg: *p* = 0.002), and IL-6 was significantly lower in both P-MCP groups at all time points with a maximum difference at 24 h post-CLP (400 mg: *p* = 0.015; 1200 mg: *p* = 0.02). In the Gal-3 inhibitor groups, 7-day mortality was significantly reduced from 61% in the control group to 28% (400 mg P-MCP: *p* = 0.03) and 22% (1200 mg P-MCP: *p* = 0.001). Rates of AKI per RIFLE criteria were significantly reduced from 89% in the control group to 44% in both P-MCP groups (400 mg: *p* = 0.007; 1200 mg: *p* = 0.007).

**Conclusions:**

This translational study demonstrates the importance of Gal-3 in the pathogenesis of S-AKI, and its potential utility as a therapeutic target.

**Graphic abstract:**

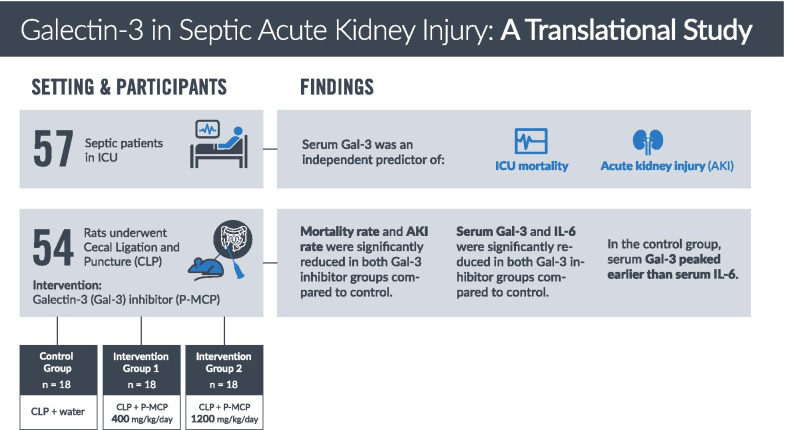

## Introduction

Sepsis-associated acute kidney injury (S-AKI) is common among critically ill patients [1–4]. S-AKI is associated with an increased risk of Intensive Care Unit (ICU) or hospital mortality [5, 6]. Further, the individual syndromes of sepsis and AKI each render a patient more vulnerable to the other [7]. Available evidence does not suggest that standard renal replacement therapies improve outcomes beyond control of fluid balance and azotemia [8]. Thus, novel approaches are necessary to prevent and treat S-AKI.

Sepsis and S-AKI result from a dysregulated immune response [9]. Foreign antigens bind to innate immune receptors and subsequently activate inflammasome components, ultimately leading to the elevated release of proinflammatory cytokines both systemically and locally [9]. The resultant cytokine storm is associated with increased levels of Interleukin-6 (IL-6) [10]. Multiple reports indicate that IL-6 is an excellent biomarker of severity and a prognostic indicator in patients with sepsis [11–15]. Findings pertaining to the therapeutic utility of IL-6 inhibition in S-AKI remain mixed [16, 17], suggesting that processes upstream of IL-6 may be responsible for triggering the deleterious effects in sepsis and S-AKI.

Galectin-3 (Gal-3) is a pleiotropic glycan-binding protein involved in numerous physiological and pathological events [18], including those that relate to immune function [19]. Epidemiologic studies have found an association between serum Gal-3 level and risk of sepsis [20–22] as well as development of chronic kidney disease (CKD) [23–25]. Pertinent findings show that serum Gal-3 predicts 30-day all-cause mortality in sepsis [26], preoperative serum Gal-3 predicts AKI after cardiac surgery [27], and serum Gal-3 at ICU discharge is associated with severity of AKI [28]. However, no previous study has examined the relationship between serum Gal-3 at the time of admission and the subsequent development of AKI in patients with sepsis. Additionally, the temporal relationship between Gal-3 and IL-6 in the pathophysiology of S-AKI has never before been explored.

Prior murine studies have demonstrated the importance of Gal-3 in the pathogenesis of sepsis [29, 30] and kidney disease [31, 32], including AKI [28, 33–36]. Pharmacologic inhibition of Gal-3 has ameliorated nephropathy induced by renal ischemia–reperfusion (IR) [28], unilateral ureteral obstruction [29], folic acid [34], hypertension [37, 38], aldosterone [39], unilateral nephrectomy [40], obesity [41], aortic stenosis [41], and cisplatin [42]. However, no murine experiment has evaluated the relationship between Gal-3 and AKI in sepsis.

Here, we present a translational study of serum Gal-3 in sepsis and S-AKI. We aimed to explore the role of Gal-3 in the pathophysiology of S-AKI and its potential utility as a therapeutic target. We examined patients admitted to the ICU with sepsis and determined whether serum Gal-3 levels predicted subsequent development of AKI and ICU mortality. In a rat model of sepsis induced by cecal ligation and puncture (CLP), we evaluated the role of Gal-3 in the pathogenesis of S-AKI, as well as the potential utility of Gal-3 as a therapeutic target. We studied the effect of an oral Gal-3 inhibitor, modified citrus pectin, on S-AKI occurrence, mortality, and levels of serum Gal-3, IL-6, and creatinine, as well as the temporal relationship between the rise of serum Gal-3 and IL-6.

## Methods

### Patient study

A prospective observational study was conducted with the approval of the Ethics Committee of Zhongnan Hospital of Wuhan University at the general ICU of Zhongnan Hospital of Wuhan University, Hubei Province, China. From January 1, 2019 to October 31, 2019, consecutive patients were enrolled following a diagnosis of sepsis according to the Third International Consensus (Sepsis-3) Definitions [43]. Patients with pre-existing AKI, CKD, renal replacement therapy, end-stage renal disease, malignancy, or organ transplantation were excluded from the study. Patients without a consent form, less than 18 years old, or over 80 years old were also excluded. An acute physiology and chronic health evaluation (APACHE II) assessment was also performed [44]. The two primary outcomes were survival in the ICU and development of subsequent AKI.

#### Serum biochemical measurements

A blood sample was obtained from patients within 6 hours (h) of admission to the ICU. Serum Gal-3 was measured by the human Gal-3 enzyme-linked immunosorbent assay (ELISA) Kit (BG Medicine, Corgenix, Inc., Broomfield, CO, USA; detection range: 1.4 ng/ml to 94.8 ng/ml), and serum creatinine (Cr) was measured using AU5831 Clinical Chemistry Analyzers (Beckman Coulter, Inc. Brea, CA, USA). In addition, serum neutrophil gelatinase-associated lipocalin (NGAL) and cystatin C (CysC) were measured with ELISA kits (Proteintech, Rosemont, IL, USA) according to the manufacturer`s instructions. Procalcitonin (PCT) was also measured using the ELFA (Enzyme-Linked Fluorescent Assay) technique by VIDAS^®^ B•R•A•H•M•S PCT™ (bioMérieux, Inc., Marcy-l'Étoile, France).

#### Evaluation of renal function and survival

AKI was evaluated according to Kidney Disease: Improving Global Outcomes (KDIGO) clinical practice guidelines based on serum Cr criteria [45]. In addition, patient survival during ICU stay was evaluated.

### Animal study

Male adult (weight 400–600 g) Sprague–Dawley rats were purchased from the Center for Animal Experiment of Wuhan University. All animals were housed in individually ventilated cages and had free access to water and food. All performed procedures were previously reviewed and approved by the Animal Care and Use Committee of Wuhan University.

#### Cecal ligation and puncture model

CLP was performed with a predetermined 25% ligated length of cecum. Following ligation, the cecum was punctured twice using a 20-gauge needle inferior to the ileocecal valve. The abdomen was then closed and 20 ml/kg of prewarmed saline was administered subcutaneously as fluid resuscitation. Rats were returned to their cages and allowed food and water ad libitum.

#### Animal experimental protocol

**Fig. 1:**
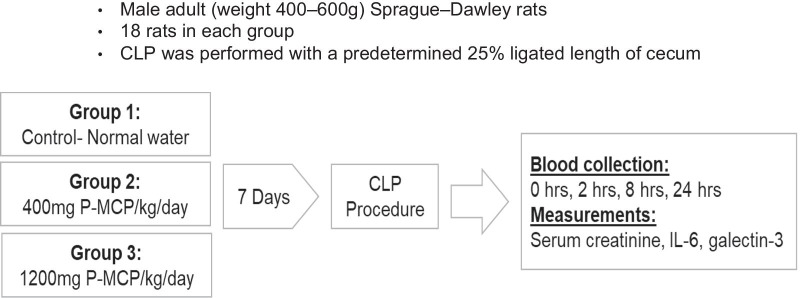
CLP experimental strategy. All rats underwent CLP. Control group (*n* = 18) received normal drinking water, while two intervention groups received a Gal-3 inhibitor, PMCP, at 400 mg/kg/d and 1200 mg/kg/d 7 d prior to CLP. Following CLP, blood was drawn at baseline, 2, 8, and 24 h post-CLP for serum biochemical measurements

The animal study included three groups (Fig. 1). All rat groups underwent CLP. Two intervention groups were administered low molecular weight modified citrus pectin (P-MCP) (PectaSol-C^®^, EcoNugenics, Santa Rosa, CA, USA) in drinking water for seven days prior to CLP. P-MCP is a dietary supplement comprised of low molecular weight pectin, which directly inhibits Gal-3 by binding to the carbohydrate recognition domain of Gal-3 [46]. The pectin is derived from the pith of citrus peels, after which it is enzymatically treated to yield pectin fibers with a molecular weight less than 15 kDa and less than 5% esterification [46]. The effect of P-MCP has been validated in multiple animal models of AKI [36, 38, 39, 41, 42].

The first intervention group (*n* = 18) received P-MCP at 400 mg/kg/day, while the second intervention group (*n* = 18) received P-MCP at 1200 mg/kg/day. The control group (*n* = 18) was not provided with P-MCP prior to CLP and received water ad libitum. P-MCP was not provided to any group following the CLP procedure.

#### Serum biochemical measurements

Blood (1.0 ml) was drawn from the jugular vein at 24 h prior to CLP (as baseline), and at 2, 8, and 24 h post-CLP. Serum was collected using EDTA as an anticoagulant. Samples were centrifuged for 15 min at 1000 × *g* at 2–8 °C within 30 min of collection. The supernatant was collected for assaying.

Serum Gal-3 levels were measured at baseline, 2, 8, and 24 h post-CLP, using an enzyme-linked immunosorbent assay (ELISA) (Lifespan Biosciences, Seattle, WA, USA; detection range: 0.156 ng/ml to 10 ng/ml)**,** and serum IL-6 level was measured at baseline, 2, 8, and 24 h post-CLP, using an ELISA (R&D Systems, Minneapolis, MN, USA). Additionally, rat serum Cr levels were measured to assess renal function using a Cr assay kit (Nanjing Jiancheng Bioengineering Institute, Nanjing, China).

#### Evaluation of renal function and survival rate

Serum Cr was measured at baseline, 2, 8, and 24 h post-CLP to assess renal function using a Cr enzymatic assay kit. AKI occurrence was determined by Risk, Injury, Failure, Loss of kidney function, and End-stage kidney disease (RIFLE) criteria at 24 h post-CLP. Per RIFLE criteria, AKI was defined as Cr level at least 150% of baseline at 24 h, and severity was categorized as risk (RIFLE-R), injury (RIFLE-I), and failure (RIFLE-F) according to Cr level at 150%, 200%, and 300% of baseline respectively at 24 h [47, 48]. Survival was evaluated at 7 days post-CLP.

#### Statistical analysis

All numerical data were expressed as mean ± standard error of the mean (SEM) or median and interquartile range. Statistical *p* < 0.05 was considered significant. Sample means were compared between groups using two-tailed t-tests and analysis of variance (ANOVA), and within-group differences were analyzed using the Wilcoxon signed-rank test. Additionally, Pearson’s chi-squared test was used to evaluate for associations between categorical variables. Survival analysis was conducted using Kaplan–Meier analysis, and mortality in each group was compared using the log-rank test.

Multivariate logistic regression was performed to analyze Gal-3 as an independent predictor of death with and without controlling for age, AKI, and APACHE II score. Multivariate logistic regression was also used to evaluate Gal-3 as an independent predictor of AKI with and without adjusting for age and APACHE II score. Area under the receiver operating characteristic curve (AUC-ROC) was estimated to evaluate the performance of Gal-3 as a predictor of S-AKI and death in ICU patients [49]. Statistical analyses were performed using R software and Microsoft 365 Excel data analysis software.

## Results

### Patient characteristics

The study sample included 57 patients who were admitted to the ICU with a diagnosis of sepsis. Table 1 summarizes patient characteristics at the time of admission and mean serum Gal-3 levels, as well as additional serum biomarkers CysC, NGAL, and PCT levels. There was no significant difference in mean age between patients that developed S-AKI and patients that did not develop S-AKI following ICU admission (age: 60.6 ± 1.6 years vs. 59 ± 2.0 years, *p* = 0.53). APACHE II score was significantly higher among patients that developed S-AKI as compared to patients that did not (18.6 ± 1.2 vs 15.2 ± 1.1, *p* < 0.05).

**Table 1:** Characteristics of patients at ICU admission

Variable	All patients (*n* = 57)	Patients without subsequent AKI (*n* = 30)	Patients with subsequent AKI (*n* = 27)	*P *value
Age (years)	59 (53–66)	59 (53–66.8)	61 (55–66)	0.53
Female sex, *n* (%)	32 (56)	18 (60)	14 (52)	0.73
ICU stay (days)	13 (9–15)	11 (8.3–13)	15 (11.5–19.5)	< 0.001
APACHE II score	16 (12–21)	14 (11–17)	20 (13.5–23)	< 0.05
Serum Gal-3 (ng/ml)	5.7 (3.6–8.9)	4.2 (3.5–7)	7.9 (4.7–16.6)	0.002
Serum CysC (μg/ml)	1.5 (1.1–2.2)	1.2 (0.9–1.5)	2 (1.7–4.1)	0.002
Serum NGAL (ng/ml)	302.9 (175.3–514.2)	243.4 (170.6–421.9)	430 (215–561)	0.406
Serum PCT (ng/ml)	11.2 (3.1–52.4)	7.7 (2.9–27.3)	32.6 (3.7–91)	0.007

Characteristics of patients in AKI and non-AKI groups. Characteristics were compared using chi-square tests or two-tailed t-tests. *AKI* acute kidney injury, *APACHE II* acute physiology and chronic health evaluation II, *CysC* cystatin C, *Gal-3* galectin-3, *ICU* intensive care unit, *NGAL* neutrophil gelatinase-associated lipocalin, *PCT* procalcitonin

#### Serum Galectin-3, AKI, and survival

**Fig. 2:**
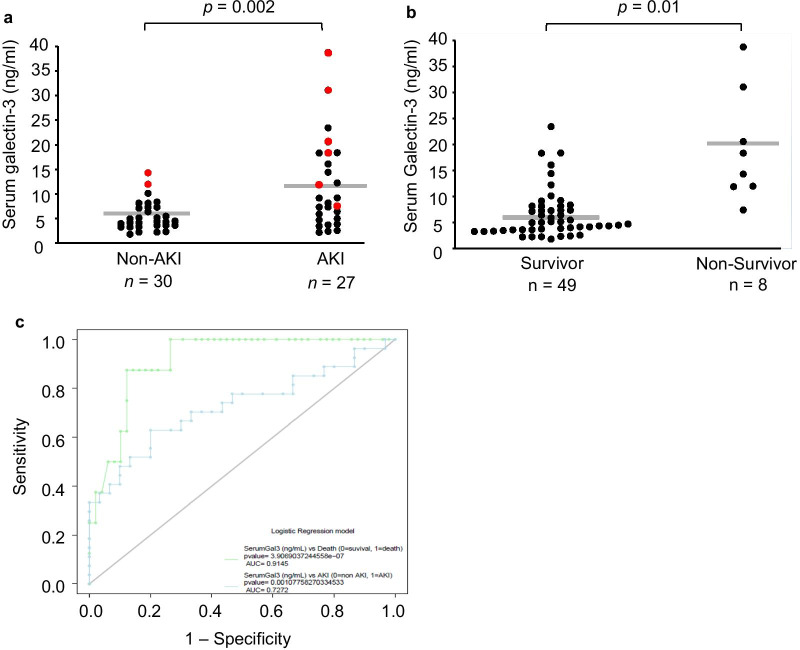
Serum galectin-3 levels at ICU admission predict subsequent acute kidney injury and ICU mortality among patients. Means are shown by the *gray horizontal bars* and displayed numerically above the corresponding columns. **a** Serum galectin-3 levels in AKI vs non-AKI groups following ICU admission (11.2 ± 1.6 ng/ml vs. 5.3 ± 0.5 ng/ml, *p* = 0.002). Red dots represent data from non-survivors, while black dots represent data from survivors. **b** Serum galectin-3 levels in survivor vs non-survivor groups following ICU admission (18.7 ± 3.6 ng/ml vs. 6.4 ± 0.6 ng/ml, *p* = 0.0002). **c** Area under the receiver operating characteristic curves for serum galectin-3 in survival (green curve) and galectin-3 in AKI (blue curve)

Of the 57 patients admitted to the ICU, 27 (47%) subsequently developed S-AKI. Additionally, 8 patients (14%) died during the ICU stay: 6 were in the S-AKI group and 2 were in the non-AKI group. Mean serum Gal-3 level was significantly higher among patients that developed subsequent S-AKI as compared to patients that did not develop S-AKI (11.2 ± 1.6 ng/ml vs. 5.3 ± 0.5 ng/ml; *p* = 0.002) (Fig. 2a). Mean serum Gal-3 level was also significantly higher among patients that died as compared to patients that did not die (18.7 ± 3.6 ng/ml vs. 6.4 ± 0.7 ng/ml; *p* = 0.01) (Fig. 2b). Using multivariate logistic regression, serum Gal-3 was associated with an increased odds of death before and after adjusting for age, AKI occurrence, and APACHE II score (OR = 1.4 [95% CI 1.1–2.2], *p* = 0.04). Serum Gal-3 was also associated with an increased odds of AKI before and after adjusting for age and APACHE II score (OR = 1.2 [95% CI 1.1–1.4], *p* = 0.01). The AUC-ROC for serum Gal-3 predicting subsequent AKI was 0.73, and the AUC-ROC for serum Gal-3 predicting ICU mortality was 0.91 (Fig. 2c).

### Rat Cecal ligation and puncture model

In total, 54 animals underwent the CLP procedure: 18 rats in the control group, and 18 rats in each P-MCP-treated group.

#### Galectin-3

**Fig. 3:**
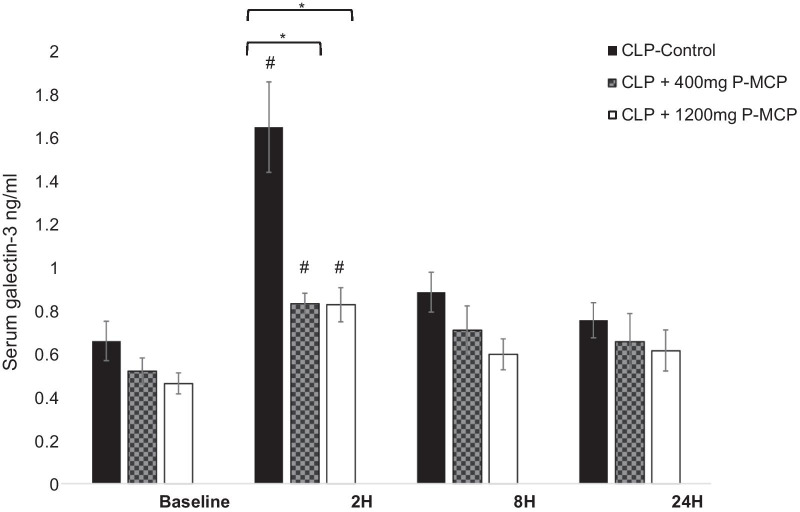
Serum galectin-3 levels with and without P-MCP pretreatment at baseline, 2 h, 8 h, and 24 h post-CLP in a rat CLP model. * indicates *p* < 0.05 for comparison between control group and P-MCP-treatment group. # indicates *p* < 0.05 for within group comparison to baseline value

**Fig. 4:**
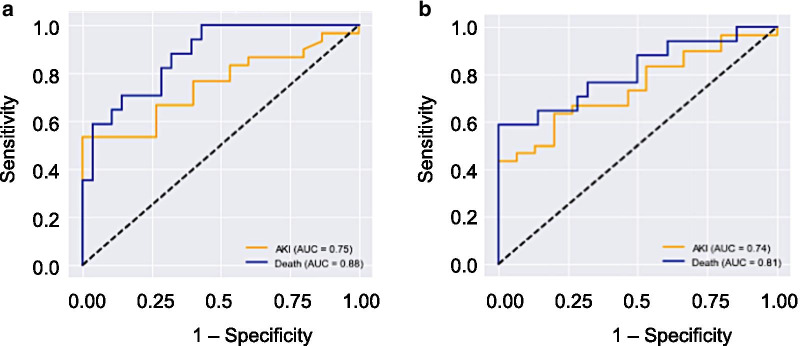
Serum galectin-3 and IL-6 predict AKI and death in a rat CLP model. **a** Area under the receiver operating characteristic curve for serum galectin-3 in AKI (yellow curve) and mortality (blue curve). **b** Area under the receiver operating characteristic curve for IL-6 in AKI (yellow curve) and mortality (blue curve)

Differences in serum Gal-3 concentrations between control and P-MCP-treated rats are shown in Fig. 3. Baseline serum values were not significantly different among the three rat groups (*F* = 3.2, *p* = 0.15). In both P-MCP-treated groups, mean serum Gal-3 levels were significantly lower than control at 2 h post procedure (400 mg P-MCP: 0.83 ± 0.05 ng/ml vs 1.63 ± 0.22 ng/ml, *p* = 0.003; 1200 mg P-MCP: 0.82 ± 0.08 ng/ml vs 1.63 ± 0.22 ng/ml, *p* = 0.001). Serum Gal-3 levels in all groups peaked at 2 h post-CLP and were significantly higher than their respective baseline values (control: *p* = 0.0001; 400 mg P-MCP: *p* = 0.001; 1200 mg P-MCP: *p* = 0.004). Gal-3 levels in all three groups subsided rapidly and were no longer significantly elevated at 8 h post CLP compared to their respective baseline values. The AUC-ROC for serum Gal-3 predicting subsequent AKI was 0.75, and the AUC-ROC for serum Gal-3 predicting mortality was 0.88 (Fig. 4a).

#### Interleukin-6

**Fig. 5:**
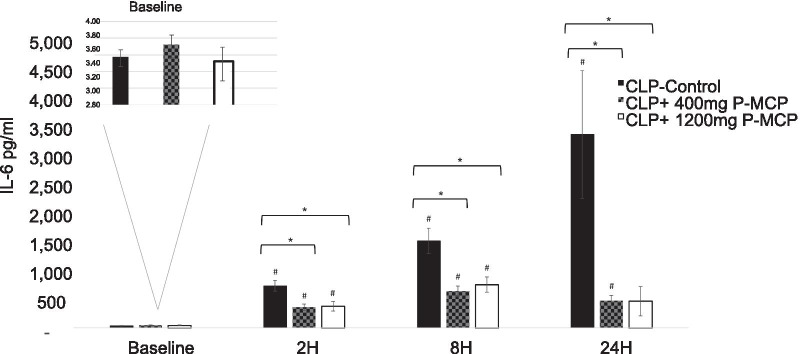
Serum IL-6 levels with and without P-MCP pretreatment in a rat CLP model. IL-6 concentrations were significantly lower in the P-MCP 400 mg and P-MCP 1200 mg group vs. the control group. * indicates *p* < 0.05 for comparison between control group and P-MCP-treatment group. # indicates *p* < 0.05 for within group comparison to baseline value

Differences in serum IL-6 concentrations between control and P-MCP-treated rats are shown in Fig. 5. Baseline values were not significantly different among the three groups (F = 3.2, *p* = 0.56). Concentrations of serum IL-6 in both P-MCP-treated groups were significantly lower than control values at all post-CLP time points, with the maximum difference at 24 h post-CLP (400 mg P-MCP: 468.0 ± 89.5 pg/ml vs 1344.5 ± 1103.4 pg/ml, *p* = 0.015; 1200 mg P-MCP: 458.8 ± 251.9 pg/ml vs 1344.5 ± 1103.4, *p* = 0.02) (Fig. 5). Serum IL-6 in the control group increased over time and peaked at 24 h post-CLP (3,344.5 ± 1,103.4 pg/ml). In both Gal-3 inhibitor groups, IL-6 levels decreased between 8 and 24 h post-procedure. The reduction in IL-6 levels between 8 and 24 h post-CLP was statistically significant in the 1200 mg P-MCP group only (631 [IQR 494.7–828.2] pg/ml vs 187.2 [IQR 156.5–272.6] pg/ml, *p* = 0.006). The AUC-ROC for serum IL-6 predicting subsequent AKI was 0.74, and the AUC-ROC for serum IL-6 predicting ICU mortality was 0.81 (Fig. 4b).

#### Creatinine

**Fig. 6:**
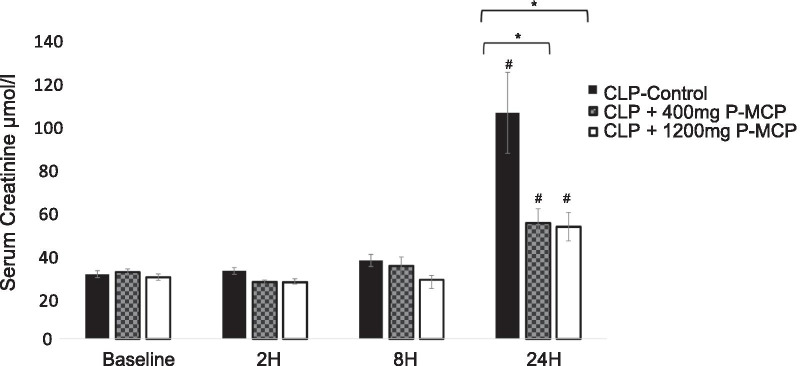
Serum creatinine levels with and without P-MCP pretreatment in a rat CLP model. * indicates *p* < 0.05 for comparison between control group and P-MCP-treatment group. # indicates *p* < 0.05 for within group comparison to baseline value

Differences in serum Cr concentrations between control and P-MCP-treated rats are shown in Fig. 6. Baseline values were not significantly different between the three groups (F = 3.2, *p* = 0.54). Serum Cr concentration in the 1200 mg P-MCP-treated group was significantly lower than control at all time points post-CLP, reaching a peak difference at 24 h post-procedure (51.8 ± 6.7 µmol/l vs 104.6 ± 18.8 µmol/l, *p* = 0.016). Serum Cr concentration in the 400 mg P-MCP-treated group was significantly lower than control group at 2 h and 24 h post-CLP (2 h: 26.3 ± 0.9 µmol/l vs 31.3 ± 1.6 µmol/l, *p* = 0.009; 24 h: 53.5 ± 4.2 µmol/l vs 104.6 ± 18.8 µmol/l, *p* = 0.005).

#### AKI

**Fig. 7:**
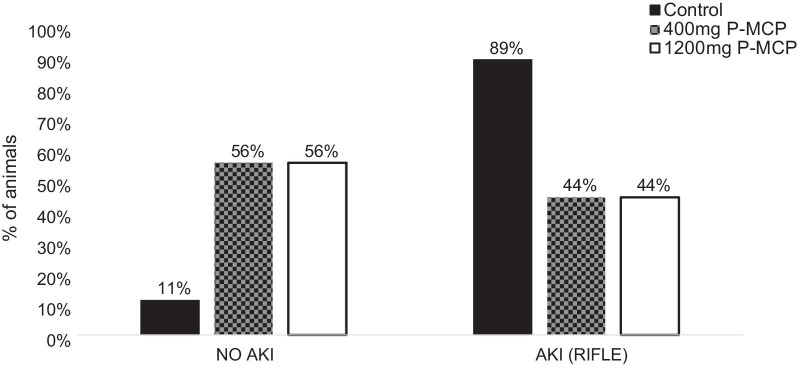
Acute kidney injury occurrence with and without P-MCP pretreatment in a rat CLP model. Percent AKI with or without P-MCP pretreatment per RIFLE criteria. Within the control group, 16 (89%) rats developed AKI. In each P-MCP group (400 mg and 1200 mg), 8 (44%) rats developed AKI

The percent of animals that developed AKI at 24 h post-CLP in the control and P-MCP-treated groups are shown in Fig. 7. As determined by RIFLE criteria, 89% (16) of rats in the control group developed AKI, while 44% (8) of rats developed AKI in the 400 mg P-MCP group and 44% (8) developed AKI in the 1200 mg P-MCP group. There was a statistically significant difference in AKI rate between the control group and both the 400 mg P-MCP group (*p* = 0.007) and 1200 mg P-MCP group (*p* = 0.007). In the control group, 8 (44%) rats were classified as RIFLE-R, 2 (11%) rats were RIFLE-I, and 6 (33%) rats were RIFLE-F. In the 400 mg P-MCP group, 1 (6%) rat was classified as RIFLE-R, 4 (22%) rats were RIFLE-I, and 3 (17%) rats were RIFLE-F. In the 1200 mg P-MCP group, 1 (6%) rat was classified as RIFLE-R, 4 (22%) rats were RIFLE-I, and 3 (17%) rats were RIFLE-F.

#### Survival

**Fig. 8:**
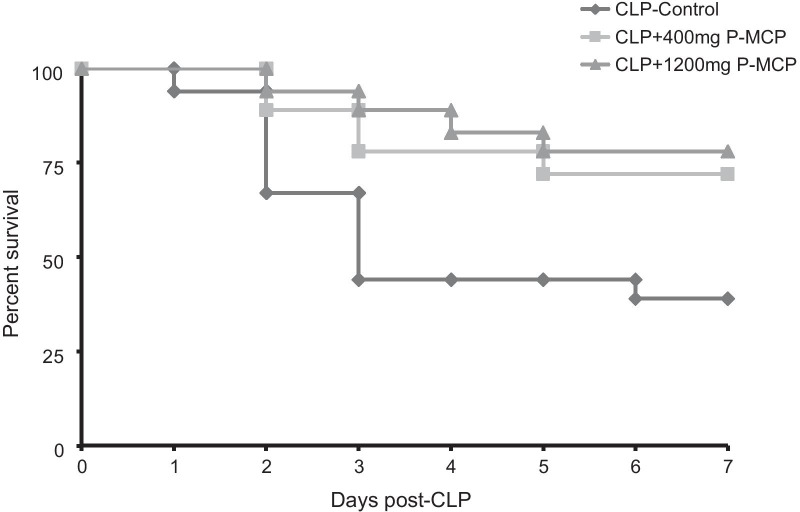
Survival curves with and without P-MCP pretreatment in a rat CLP model. Each group started with 18 rats prior to CLP procedure. By day 7, 11 (61%) rats had died in the control group, compared to only 5 (28%) rats in the 400 mg P-MCP group (*p* = 0.03) and 4 (22%) rats in the 1200 mg P-MCP group (*p* = 0.001)

Survival data are shown in Fig. 8. Each group began with 18 rats prior to CLP. At 7 days post-CLP, 11 (61%) rats had died in the control group compared to 5 (28%) rats in the 400 mg P-MCP group and 4 (22%) rats in the 1200 mg P-MCP group. Mortality was significantly lower in both P-MCP treated groups as compared to controls (400 mg P-MCP: *p* = 0.03; 1200 mg P-MCP: *p* = 0.001).

## Discussion

In this translational study, we evaluated the role of Gal-3 in sepsis and S-AKI. We found that elevated Gal-3 levels at ICU admission predicted S-AKI and mortality in patients with sepsis, while inhibition of Gal-3 in a CLP rat model resulted in a statistically significant reduction in S-AKI and mortality. Together, these findings suggest that Gal-3 plays an important role in the pathophysiology of S-AKI and sepsis mortality.

In patients admitted to the ICU with sepsis, elevated serum Gal-3 levels at admission predicted ICU mortality. Our findings are consistent with other clinical research in which serum Gal-3 levels predicted 30-day all-cause mortality in sepsis [26], as well as murine experiments demonstrating the important role of Gal-3 in sepsis pathogenesis and mortality [27, 30]. Our findings suggest that serum Gal-3 may serve as a prognostic indicator in sepsis.

We also found that serum Gal-3 levels obtained at ICU admission were associated with subsequent AKI, as were two previously studied biomarkers of kidney injury, CysC and PCT [50–52]. Further, Gal-3 levels predicted subsequent development of S-AKI after controlling for age and APACHE II score. This is the first report of such a finding in patients with sepsis. It is consistent with previous findings that preoperative serum Gal-3 predicts AKI after cardiac surgery [27], and that Gal-3 at the time of ICU discharge is associated with severity of AKI [28]. Our findings are also consistent with various murine experiments, which demonstrate that Gal-3 plays a role in the pathophysiology of AKI secondary to various insults [28, 33–36]. In our patient study, 6 of the 8 observed deaths were in patients who had developed S-AKI, suggesting that the increased mortality seen in those with high Gal-3 levels may be mediated, at least in part, by impaired renal function [53]. While there was an association between Gal-3 and AKI, the predictive ability of Gal-3 in the detection of AKI was lower than that of mortality. Notably, the patient study was limited by its observational nature, small sample size, and low mortality rate. The findings demonstrate an association of Gal-3 with both mortality and AKI, and warrant further evaluation of the role of Gal-3 in S-AKI and sepsis mortality.

In a CLP rat model, we found that administration of a Gal-3 inhibitor prior to CLP significantly reduced mortality and S-AKI rate in both P-MCP-treated groups. Inhibition of Gal-3 also resulted in a significant reduction in Gal-3 and IL-6 levels. The difference between IL-6 levels in the control and P-MCP-treated groups increased over time, reaching the greatest difference at 24 h post-CLP. Prior studies have also reported attenuation of IL-6 levels with Gal-3 knock out or Gal-3 inhibition [28, 29, 35, 37].

In the control group, serum Gal-3 levels peaked earlier than serum IL-6: Gal-3 levels peaked at 2 h post-CLP and fell to near-baseline by 8 h, while IL-6 continued to rise throughout the entire 24 h post-procedure period. Comparatively, in the Gal-3 inhibitor groups, IL-6 levels decreased between 8 and 24 h post-procedure. Compared to controls, the decrease in IL-6 between 8 and 24 h was statistically significant in the 1200 mg P-MCP group only. Together, these findings suggest that serum Gal-3 release may precede the rise of IL-6 in the inflammatory cascade. Additionally, P-MCP-mediated Gal-3 inhibition may exert its effect in a dose dependent manner. At higher doses, Gal-3 inhibition may cause IL-6 levels to drop at an earlier time point.

Given the important role of IL-6 in the inflammatory cascade, these preliminary findings suggest that Gal-3 may serve as an upstream mediator of the “cytokine storm” in sepsis and S-AKI [54]. Study limitations included small sample sizes, as well as limited follow-up time to monitor Cr rise. Additionally, given our aim to compare mortality among CLP groups, histological evaluation of renal tissue was not possible.

In humans, elevations in serum Gal-3 may persist for longer durations. In a study by Prud’homme et al*.*, 645 patients with AKI during their ICU stay demonstrated elevated serum Gal-3 levels at discharge [28]. Further, Gal-3 levels at discharge were increasingly elevated with increased severity of AKI [28]. Given these findings, Gal-3 may continue to play a role in the progression of AKI past the 2 to 8 h range detected in our rat model. Our findings warrant further investigation of the potential therapeutic utility of Gal-3 inhibition or removal in the prevention and treatment of S-AKI.

## Conclusion

This translational study demonstrates the important role of Gal-3 in the pathogenesis of S-AKI, as well as its potential utility as a therapeutic target. Further studies, including randomized trials, are warranted to examine the role of Gal-3 as a therapeutic target in the treatment of sepsis and S-AKI.

## Data Availability

The datasets used and/or analyzed during the current study are available from the corresponding author on reasonable request.

## Abbreviations

AKI: Acute kidney injury
APACHE II: Acute physiology and chronic health evaluation
AUC-ROC: Area under the receiver operating characteristic
CLP: Cecal ligation and puncture
CKD: Chronic kidney disease
Cr: Creatinine
CysC: Cystatin C
ELFA: Enzyme-linked fluorescent assay
ELISA: Enzyme-linked immunosorbent assay
Gal-3: Galectin-3
IL-6: Interleukin-6
ICU: Intensive care unit
KDIGO: Kidney disease: improving global outcomes
P-MCP: Pectasol modified citrus pectin
NGAL: Neutrophil gelatinase-associated lipocalin
PCT: Procalcitonin
RIFLE: Risk, injury, failure, loss of kidney function, and end-stage kidney disease
S-AKI: Sepsis-associated AKI
